# Substantial Improvement of Short Wavelength Response in n-SiNW/PEDOT:PSS Solar Cell

**DOI:** 10.1186/s11671-015-0998-9

**Published:** 2015-08-19

**Authors:** Zhaoyun Ge, Ling Xu, Yunqing Cao, Tao Wu, Hucheng Song, Zhongyuan Ma, Jun Xu, Kunji Chen

**Affiliations:** School of Electronic Science and Engineering and Collaborative Innovation Center of Advanced Microstructures of National Laboratory of Solid State Microstructures, Nanjing University, Nanjing, 210093 People’s Republic of China; College of Mathematics and Physics Science, Jiangsu University of Science and Technology, Zhenjiang, 212003, Jiangsu Province People’s Republic of China

**Keywords:** Si nanowire (SiNW), Hybrid solar cell, External quantum efficiency, PCE

## Abstract

We report herein on the effects of silicon nanowire with different morphology on the device performance of n-SiNW/PEDOT:PSS hybrid solar cells. The power conversion efficiency (PCE) and external quantum efficiency (EQE) of the SiNW/PEDOT:PSS hybrid solar cells can be optimized by varying the length of the silicon nanowires. The optimal length of silicon nanowires is 0.23 μm, and the hybrid solar cell with the optimal length has the *V*_oc_ of 569 mV, *J*_sc_ of 30.1 mA/cm^2^, and PCE of 9.3 %. We fabricated more isolated silicon nanowires with the diluted etching solution. And the *J*_sc_ of the hybrid solar cell with more isolated nanowires has a significant enhancement, from 30.1 to 33.2 mA/cm^2^. The remarkable EQE in the wavelength region of 300 and 600 nm was also obtained, which are in excess of 80 %. Our work provides a simple method to substantially improve the EQE of hybrid solar cell in the short wavelength region.

## Background

Silicon is the most widely used material for solar cell production due to its abundance, nontoxicity, reliability, and mature technology. The hybrid solar cells based on silicon nanostructure and conjugate polymer poly (3,4-ethylenedioxy-thiophene):poly(styrenesulfonate) (PEDOT:PSS) have attracted much attention for their simple fabrication process and low cost compared to the conventional p-n Si solar cells, which require high temperature (~1000 °C) processing for ion implantation and dopant diffusion [1–6]. When light illuminates the surface of the solar cell, silicon absorbs solar light, and electron–hole pairs are generated and separated at the Schottky barrier of the n-Si/PEDOT:PSS heterojunction. The photogenerated electrons are transported to the cathode through n-Si, while the holes move to the anode through the PEDOT:PSS layer. The main challenge of it is that its photoelectric conversion efficiency (PCE) is not high enough to mass production. Over the past few years, many researches have been carried out by using silicon textures for light trapping characteristics [7–11], surface passivation for high PCE and good stability [12–15], etc., leading to notable efficiency improvement with PCEs ranging from 9 to 14 %.

However, the light response of the hybrid solar cell in short wavelength region is still weak. In the present paper, we mainly research the effects of different silicon nanowire length and the morphology of silicon nanowires on the light response of n-SiNW/PEDOT:PSS hybrid solar cells. The external quantum efficiency (EQE) was measured with a QEx10 at room temperature to investigate the spectral response of solar cells consisting of different morphology silicon nanowires. Scanning electron microscope (SEM) was used to observe the characterization of the silicon nanowire arrays. The density-voltage (*J*–*V*) characteristics of the solar cells were measured both in dark and under illumination by using a Keithley 610C electrometer.

## Methods

### Fabrication of SiNW Arrays

The c-Si wafer (CZ, 1.7–3.2 Ω cm, 550 μm thickness) was cleaned by the RCA1 and RCA2 procedures. The vertically aligned n-silicon nanowires were performed by being immersed into an etchant composed of HF and AgNO_3_ mixtures for an anisotropic wet-chemical etching process at room temperature [16]. In order to investigate the effect of silicon nanowire length on SiNW/PEDOT:PSS hybrid solar cell performance, the silicon nanowire length was controlled by varying the etched time. The etching time was set to 1, 2, 5, and 8 min with an etchant consisting of 5 M HF acid and 0.02 M AgNO_3_. Following nanowire fabrication, the wafer with vertically aligned SiNW arrays was rinsed with deionized water and cleaned with concentrated nitric acid for 20 min to remove all Ag dendritic structures from the nanowire surfaces.

### Fabrication Procedure of PEDOT:PSS/SiNW Solar Cells

The fabrication of SiNW/PEDOT:PSS hybrid solar cells was as follows: Firstly, the wafer with vertically aligned SiNW arrays was immersed in the solution of dilute HF for 5 min to remove the oxide layer on the SiNWs and on the back of the wafer. Then, it was dried under a stream of nitrogen gas. Using this process, defects on the SiNW surface were passivated with hydrogen. Immediately after the cleaning treatment, highly conductive PEDOT:PSS (Clevios PH1000) with 5 wt.% dimethyl sulfonate (DMSO) and 0.1 wt.% zonyl fluorosurfactant was spin coated onto the surface of silicon nanowire arrays at 3000 rpm for 2 min in air to form hybrid solar cells consisting of SiNW/PEDOT:PSS core/shell radial heterojunctions. Finally, a silver finger-grid electrode was deposited on the surface of the PEDOT:PSS by DC sputtering through a shadow mask, while a 600-nm-thick aluminum electrode was deposited by DC sputtering onto the backside of the wafer.

## Results and Discussion

### Characterization of SiNW/PEDOT:PSS Hybrid Structure

**Fig. 1:**
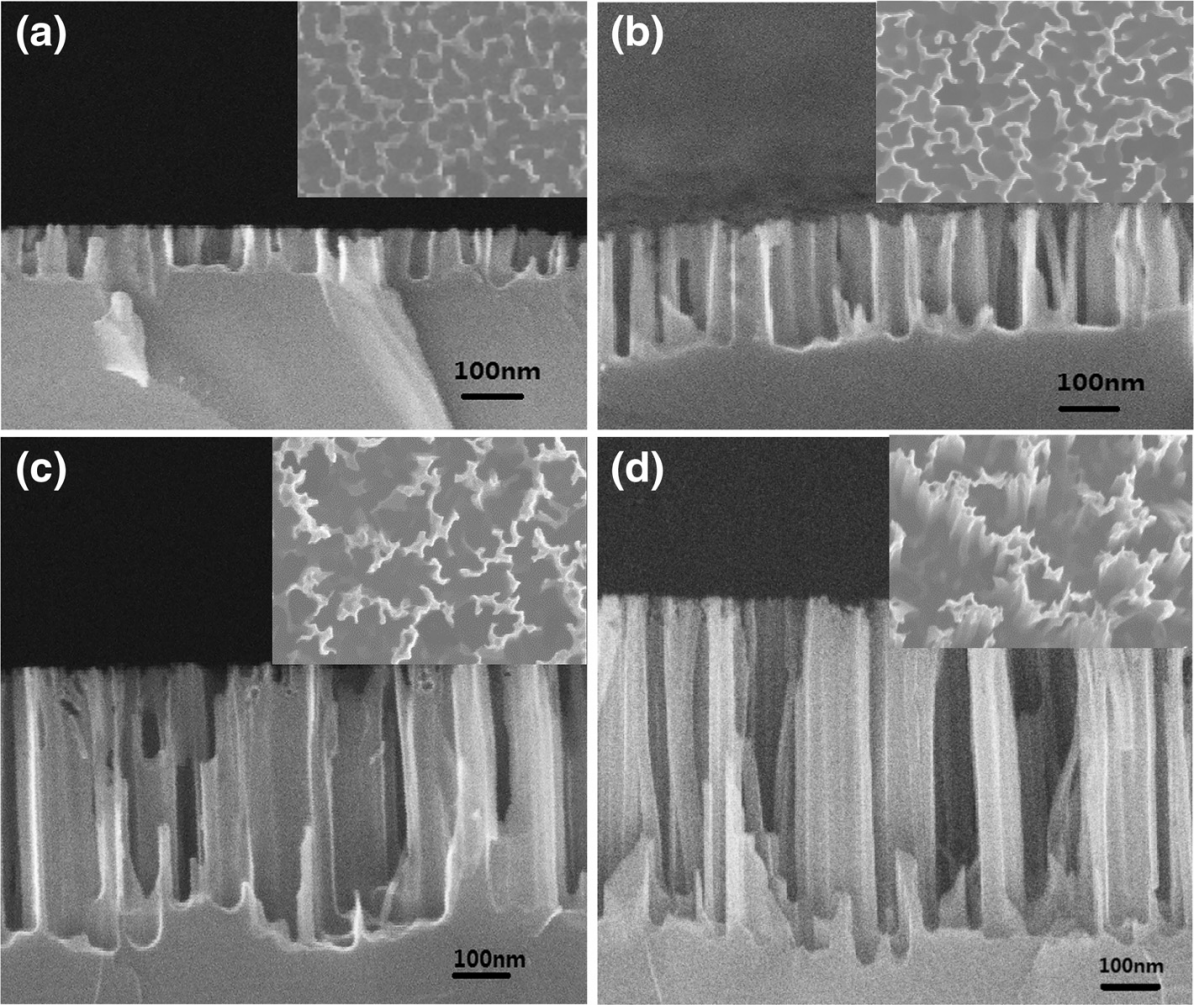
**a-d** Cross-sectional view SEM images of the SiNW with wire length (*L*) of 0.10, 0.23, 0.52, and 0.80 μm. The *inserts* show the corresponding top view SEM images of these samples

To characterize the morphology of silicon nanowire arrays with different length of the SiNW arrays, top view and cross-sectional view scanning electron microscope (SEM) analysis was employed, as shown in Fig. 1. From Fig. 1, we can see that SiNW arrays are aligned vertically over the area up to the wafer size. The etching time was set to 1, 2, 5, and 8 min with the etchant consisting of 5 M HF acid and 0.02 M AgNO_3_, resulting in nanowire lengths of 0.10, 0.23, 0.52, and 0.80 μm, respectively.

### Effects of NW Length on the Performance of SiNW/PEDOT:PSS Hybrid Solar Cells

**Fig. 2:**
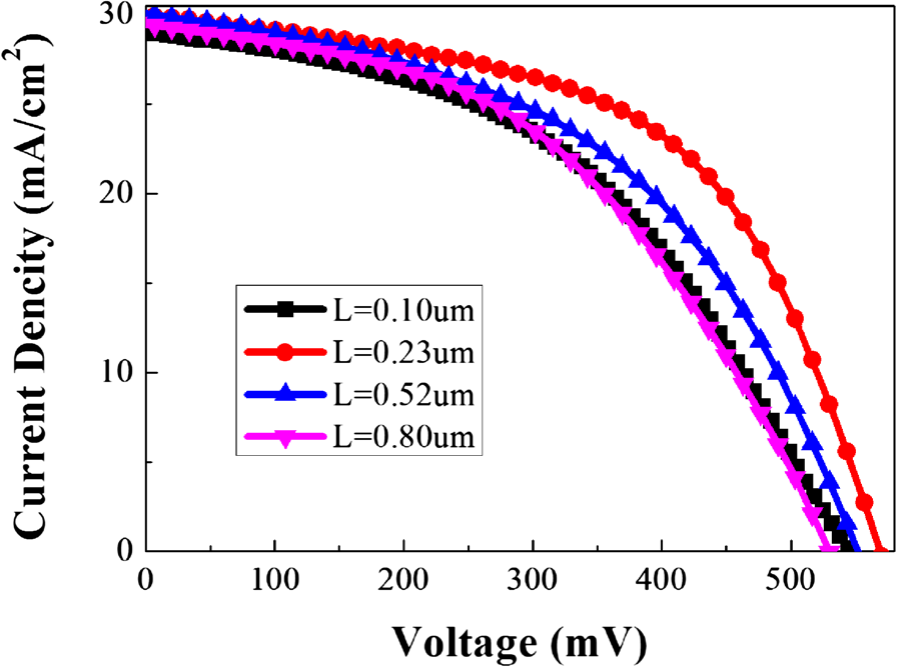
Current density-voltage characteristics of the hybrid solar cells with different lengths of silicon nanowire arrays under a simulated AM1.5G illumination condition

The current *J*–*V* characteristics of the hybrid solar cells with different lengths of SiNW arrays (shown in Fig. 2a) were measured under 100 mW/cm^2^ air mass 1.5 global (AM 1.5G) illumination. Four solar cells with different nanowire length (*L*) were investigated and the device parameters, such as short circuit current density (*J*_sc_), open circuit voltage (*V*_oc_), fill factor (FF), PCE, etc. are deduced from the *J*–*V* characteristics (summarized in Table 1). It is seen that the PCE increases with *L* and reaches a maximum of 9.3 % at *L* = 0.23 μm. It decreases to 7.2 % as *L* is further increased to 0.80 μm. The *J*_sc_ decreases from 30.1 to 29.3 mA/cm^2^ as *L* is increased from 0.23 to 0.80 μm. As seen from Table 1, the maximum value of *V*_oc_ and FF were obtained at the optimal length of *L* = 0.23 μm. As *L* is increased further to *L* = 0.80 μm, both *V*_oc_ and FF decrease monotonically to 528 mV and 46 %.

**Table 1:** The performance of hybrid solar cells with different lengths of silicon nanowire arrays

	*V*_oc_(mV)	*J*_sc_(mA/cm^2^)	FF(%)	PCE(%)	*R*_sh_(Ω cm^2^)
*L* = 0.10 μm	552	29.0	45	7.3	157
*L* = 0.23 μm	569	30.1	55	9.3	167
*L* = 0.52 μm	552	30.1	48	8.0	194
*L* = 0.80 μm	528	29.3	46	7.2	181

**Fig. 3:**
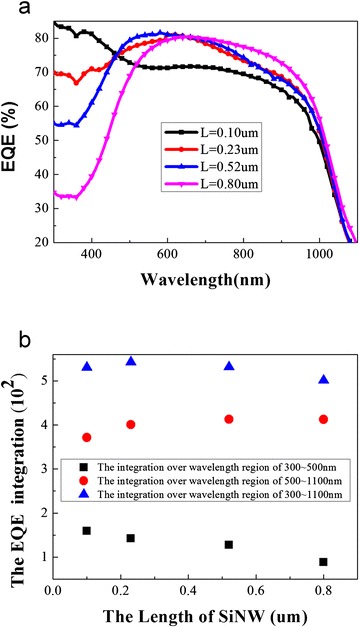
**a** The EQE of the hybrid solar cells with different *L* and **b** corresponding integration EQE over different wavelength range varies with the length of SiNW

The EQE, which is the percentage of electrons collected per incident photon, can be used as a measure of the efficiency of charge transport given that the following quantities are comparable for a set of devices: (i) incident light intensity; (ii) fraction of light absorbed; (iii) charge collection efficiency at the electrodes; and (iv) the charge transfer efficiency [17]. In order to further study the mechanism of the change of *J*_sc_, the EQE of the hybrid solar cells was measured and shown in Fig. 3a. The EQE decreases with the increase of the nanowire length in the short wavelength region (300–500 nm) but increases continuously as the length of silicon nanowires increase in the long wavelength region of 500–1100 nm. To obtain a more intuitive picture of the trend of EQE, we integrate the EQE over different wavelength ranges (shown in Fig. 3b). The optimal length for highest EQE is 0.23 μm.

We thought that the change of the EQE is mainly due to the fraction of light absorbed and the charge transfer efficiency. The conjugated polymer PEDOT:PSS is transparent and conductive (<1000 S/cm) [18, 19]. As the short wavelength light is absorbed at the PEDOT:PSS/Si interface, the photo-generated carriers were separated at the Schottky barrier of the n-Si/PEDOT:PSS heterojunction [20]. The hybrid solar cells made from nanowire arrays with shorter length have lower probability of carrier recombination due to the reduced transportation distance, resulting in the increase of the minority carrier lifetime [21, 22]. So, the EQE of the hybrid solar cell with short nanowire arrays exhibits better performance in the short wavelength.

**Fig. 4:**
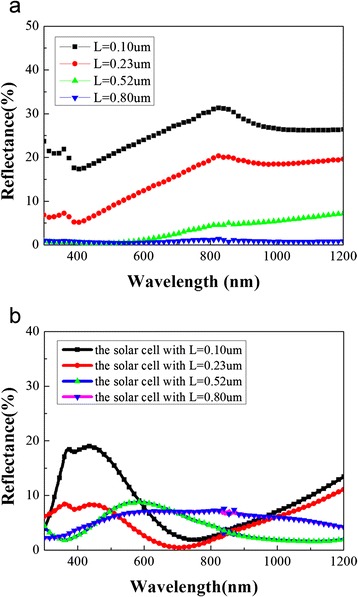
The reflectance spectra of the silicon nanowire arrays (**a**) and the hybrid solar cells (**b**) with different *L*

For our sample, the carriers generated by absorbing photon energies at the silicon nanowires diffuse to the bottom electrode and finally be collected. Hence, for longer silicon nanowires, the photon-generated carriers travel more distance before they were collected. This increases the probability of carrier recombination and therefore decreases the carrier collection efficiency of the solar cell. Besides, longer silicon nanowires have more surface area in comparison to the shorter one. We assume that the density of surface defects are the same, therefore, longer silicon nanowires have more surface defects. This will also increase the probability of carrier recombination. By contrast, the EQE spectra near the infrared region have not seen a significant change. We thought that it is mainly due to two facts: on the one hand, the longer SiNWs will have more light-trapping effect than the shorter SiNWs [22, 23]. The reflectance measurement does confirm this because the reflectance decreases with the wire’s length (as shown in Fig. 4a); on the other hand, for longer silicon nanowires with more surface area and more surface defects, carriers must diffuse and travel more distance through the silicon nanowire, therefore the fewer numbers of carrier were collected in comparison to that of shorter silicon nanowire [24]. Due to the decrease of both reflectance and the collection efficiency of carriers, the EQE spectra near the infrared region have no significant change with different lengths of SiNWs.

To further investigate the absorption of the silicon nanowire with different length, we also measured the reflectance of the silicon nanowire arrays with a different *L* coated with and without PEDOT:PSS using a UV/visible/NIR spectrophotometer (shown in Fig. 4a, b). It is seen that the reflectance of the silicon nanowire decreases with the increase of the length of the silicon nanowire arrays. The reflectance of the cells with *L* = 0.10 and 0.23 μm is higher than other cells in the short wavelength region. Compared to the EQE and the reflectance of the silicon nanowire coated with PEDOT:PSS, We postulate that this might be due to the fact that PEDOT:PSS layer acts as an antireflective coating and then substantially reduces the reflectance of light. This antireflective effect may be stronger than the light-trapping effect arising from the increase of the silicon nanowire arrays, which can also be deduced from the EQE spectra—the EQE in the hybrid solar cell with shorter nanowire arrays is higher than the cell with longer nanowire arrays in the short wavelength region.

The *V*_oc_ shows maximum values of 569 mV at *L* = 0.23 μm. It may be related to the increase in shunt resistance (*R*_sh_) with the increase of the length. The increase in the *R*_sh_ enables the high shutting power losses in hybrid solar cells, causing the enhancement in the *V*_oc_ [22]. The increase in the minority carrier lifetime can be considered as the other reason for higher *V*_oc_. The hybrid solar cells with shorter silicon nanowire length have lower probability of carrier recombination due to the reduced transportation distance, resulting in the increase of the minority carrier lifetime. Due to the increase of both *R*_sh_ and minority carrier lifetime, the 0.23-μm SiNW/PEDOT:PSS hybrid solar cell attains the highest *V*_oc_.

**Fig. 5:**
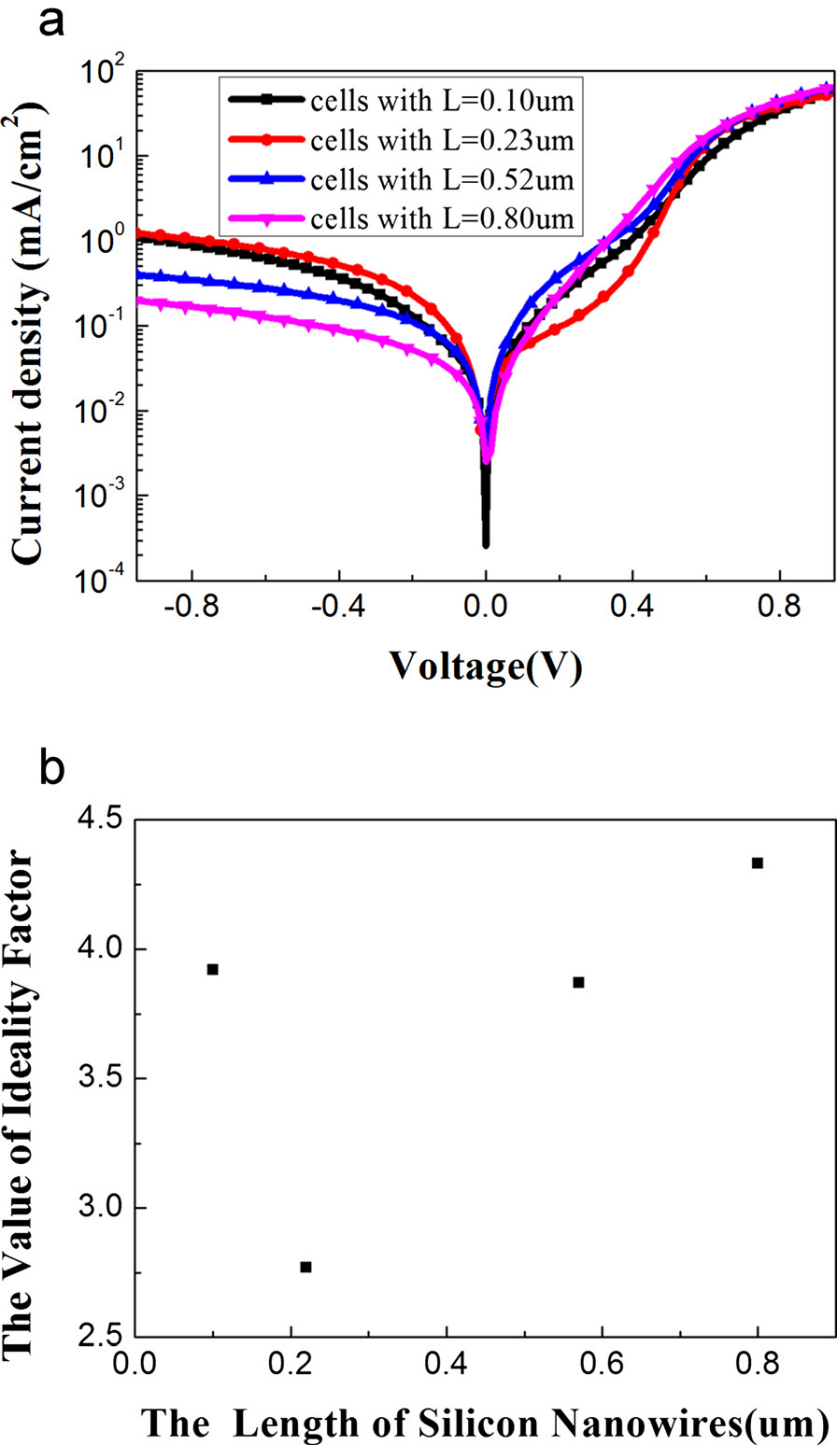
**a** The dark current density-voltage (*J*–*V*) characteristics of the silicon nanowire hybrid solar cells and **b** the corresponding values of the ideality factor which is fitted by the *dark J*–*V curves* of the devices

To further investigate the effect of different length silicon nanowire arrays, we also measured the dark current *J*–*V* characteristics of the hybrid solar cells, which are shown in Fig. 5a. The dark *J*–*V* curves of all the samples exhibit rectification characteristic. The *J*–*V* characteristic of the Schottky junction can be expressed by the thermionic emission model by Eq. 1,

1 $$ J={J}_S\left( \exp \left(\frac{qV}{n{k}_BT}\right)-1\right) $$

where *J*_*S*_ is the reverse saturation current density, *n* is the diode ideality factor, *k*_*B*_ is the Boltzmann constant (1.38 × 10 ^−23^ m ^2^ kg s ^−2^ K^−1^), *T* is the absolute temperature (298 K), and *q* is the electronic charge (1.6 × 10^−19^ °C). The ideality factor is estimated and exhibited in Fig. 5b. It can be seen that the ideality factors are different for the devices. The ideality factor is usually used to analyze the electronic process in Schottky diodes. The devices with *n* > 1 means that they have a high electron–hole recombination current within the depletion region. The decrease of the ideality factors suggests the reduction of the charge recombination [25]. The ideality factor of the cell with 2 min etched time has the minimum value (*n* = 2.77). This means that the charge recombination rate within the device is less than the others. The result indicates that the hybrid solar cell with 0.23-μm nanowire array has the maximum collection efficiency of the photon-generated carriers and then exhibits better photovoltaic performance.

### Effects of the Morphology of the Silicon Nanowire Arrays on SiNW/PEDOT:PSS Hybrid Solar Cell Performance

**Fig. 6:**
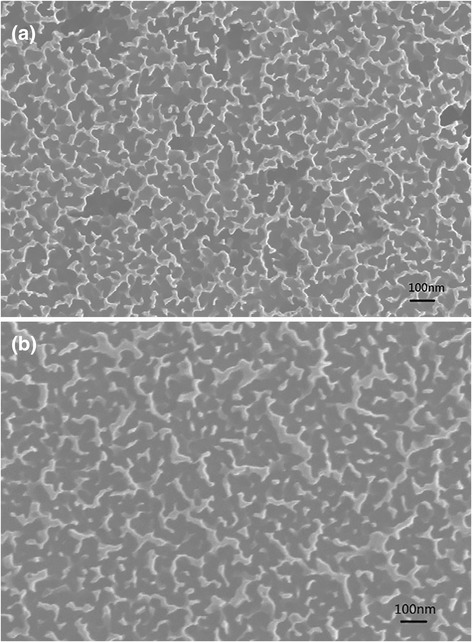
Top view SEM images of the SiNW etched with normal solution (**a**) and with diluted solution (**b**)

Based on the analysis above, it is expected that the morphology of the silicon nanowire arrays will also affect the performance of the solar cell. The etchant consisting with another concentration of 2.5 M HF acid and 0.01 M AgNO_3_ was also used to fabricate SiNW. The hybrid solar cell with SiNW of *L* = 0.23 μm etched by the diluted solution were denoted by 2, which was compared with the hybrid solar cells with *L* = 0.23 μm (denoted by 1). Top view scanning electron microscope (SEM) analysis was employed to characterize the morphology of the silicon nanowire arrays etched with different concentration solutions, as shown in Fig. 6a, b. We can find that the silicon nanowire etched with diluted solution are disconnected with each other, but the silicon nanowire etched with high concentration solution is connected.

**Fig. 7:**
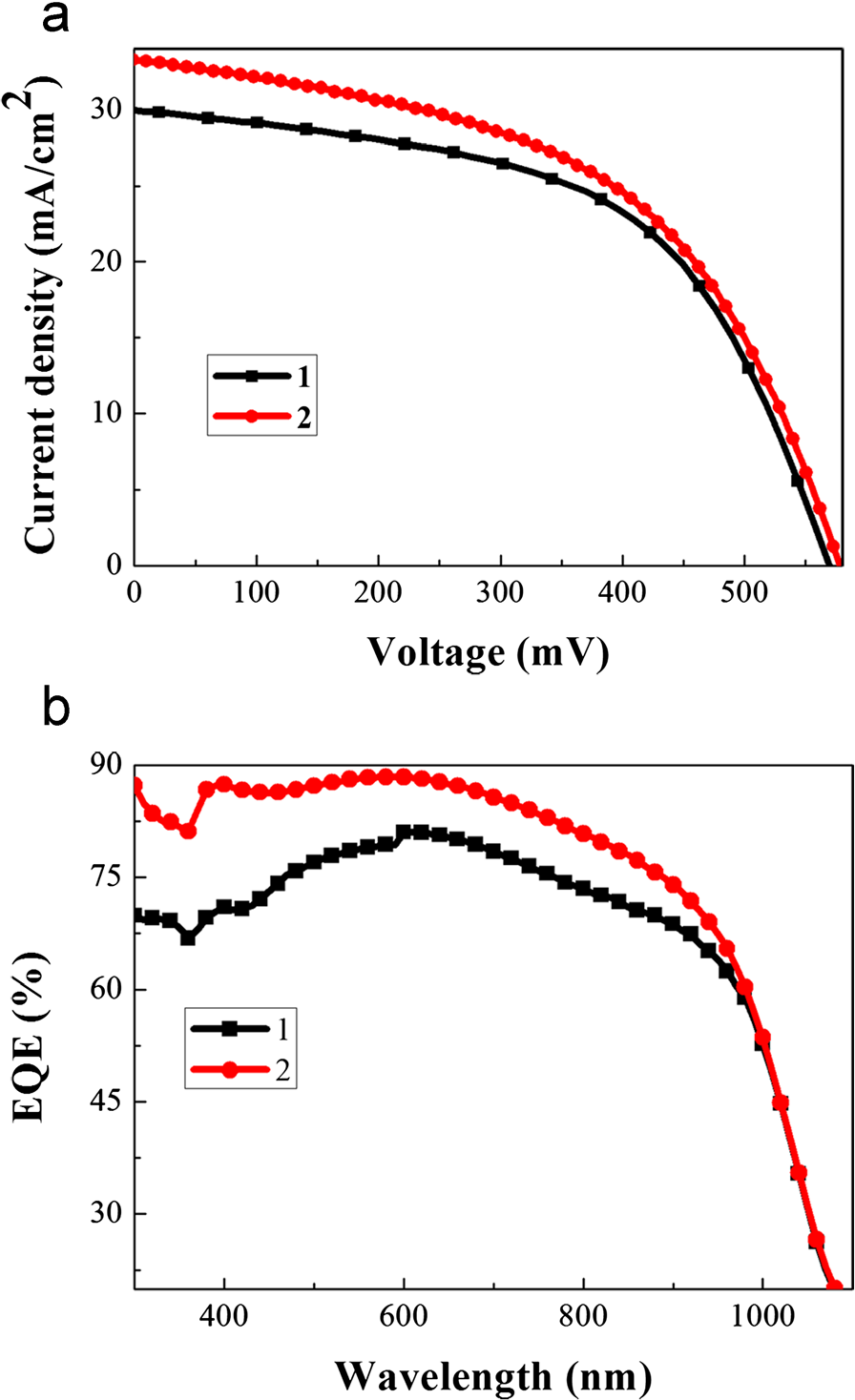
**a** Current density-voltage characteristics of the two hybrid solar cells under a simulated AM1.5G illumination condition. **b** The EQE of the two SiNW/PEDOT:PSS hybrid solar cells

Figure 7a shows the current density-voltage characteristics of hybrid solar cells etched with different concentration etchants under 100 mW/cm^2^ air mass 1.5 global (AM 1.5G) illumination. Their photovoltaic parameters of *J*_sc_, *V*_oc_, FF, and PCE, calculated from the *J*–*V* data, are summarized in Table 2. When the hybrid solar cell is made from the silicon nanowire etched by diluted solution, the *J*_sc_ has a significant enhancement, from 30.1 to 33.2 mA/cm^2^. As a result, the PCE of the cell increases from 9.3 to 9.8 %. The *V*_oc_ and FF of the hybrid solar cells with different morphology of silicon nanowires have no significant change.

**Table 2:** Performance of the hybrid solar cells

	*V*_oc_(mV)	*J*_sc_(mA/cm^2^)	FF(%)	PCE(%)
1	569	30.1	55	9.3
2	577	33.2	51	9.8

The EQE spectra of the two SiNW/PEDOT:PSS hybrid solar cells were measured to investigate the change of the *J*_sc_ (shown in Fig. 7b). The EQE is dramatically increased in the wavelength region from 300 to 600 nm when we diluted the etchant solution, which exceeds 80 %. Compared with the Sun Baoquan’s research group, our samples have a lower PCE because the solar cells they fabricated have a higher FF and a larger *V*_oc_ [26]. However, the EQE of the hybrid solar cell we fabricated is higher than that of the cell they fabricated in the short wavelength region (shown in Fig. 7b).

The reason of the enhancement of *J*_sc_ can be explained as follows: Firstly, the silicon nanowire etched with diluted solution is isolated (Fig. 6b), and the radial junction structure is easily formed. Then, these radial core-shell heterojunctions could be beneficial for charge separation and carrier collection [21], so the hybrid solar cell with isolated silicon nanowires has higher EQE; Secondly, the silicon nanowire etched with diluted solution has more space in the adjacent arrays. PEDOT:PSS will easily fill the space between SiNWs, and then the junction area is much increased. So, the *J*_sc_ of sample 2 will be enhanced [27]. Due to the radial core-shell heterojunction and the increase of the junction area, the *J*_sc_ of hybrid solar cell with isolated silicon nanowires has a significant enhancement.

## Conclusions

In summary, we designed inorganic/organic hybrid radial heterojunction solar cells combining PEDOT:PSS with vertically aligned n-type SiNWs. The PCE of the SiNW/PEDOT:PSS hybrid solar cells can be substantial enhanced by optimizing the morphology of the SiNW. Due to the reduced transportation distance of carriers in SiNWs, high carrier lifetime and carrier transport efficiency between the organic layer and the SiNWs are obtained. This improves the carrier collection efficiency in the shorter wavelength region. With the optimization of the morphology of the SiNWs, a hybrid solar cell with the SiNWs of *L* = 0.23 μm and etched by the diluted etchant solution achieves the better performance with a PCE of 9.8 %. This solar cell also has a remarkably high EQE in the wavelength region of 300–600 nm. The EQE of this hybrid solar cell in short wavelength region is in excess of 80 %. These results show that the PCE and EQE of the SiNW/PEDOT:PSS hybrid solar cells can be optimized by tuning the length of the silicon nanowires and the etching conditions during NW formation. Our approach is a significant contribution to the design of high-performance and low-cost inorganic/organic hybrid solar cell.

## Abbreviations

SiNW/PEDOT:PSS: Si nanowires/PEDOT:PSS
PCE: power conversion efficiency
EQE: external quantum efficiency
